# Eliciting Expressions of Emotion: An Exploratory Analysis of Alexithymia in Adults with Autism Utilising the APRQ

**DOI:** 10.1007/s10803-022-05508-z

**Published:** 2022-04-08

**Authors:** Christian Ryan, Stephen Cogan

**Affiliations:** 1 School of Applied Psychology, University College Cork, Distillery House, North Mall, Cork, T23 TK30 Ireland; 2 Aspect, Cork Association for Autism, Carrigtwohill, Cork, Ireland

**Keywords:** Autism spectrum disorder, Alexithymia, Linguistic analysis, Emotional expression

## Abstract

This study examined alternative methods for detecting alexithymia to the Toronto Alexithymia Scale—20 (TAS-20) by comparing the emotional linguistic performance of ASD and NT samples (n = 32 in each) on the Alexithymia Provoked Responses Questionnaire (APRQ). We utilised both the LIWC and tidytext approaches to linguistic analysis. The results indicate the ASD sample used significantly fewer affective words in response to emotionally stimulating scenarios and had less emotional granularity. Affective word use was correlated with ASD symptomatology but not with TAS-20 scores, suggesting that some elements of alexithymia are not well detected by the TAS-20 alone. The APRQ, in combination with the tidytext package, offers significant potential for sophisticated exploration of emotional expression in ASD.

When someone threatens to shoot you with a gun; when someone compliments you; when you are arrested for a crime you did not commit; when someone you love dies—what emotions do you feel? Responses to questions such as these are at the heart of the Alexithymia Provoked Responses Questionnaire (APRQ; Krystal et al., 1986). Originally developed as a proto-type self-report measure within the Beth Israel Psychosomatic Questionnaire (BIPQ; Apfel & Sifneos, 1979), the test was subsequently renamed as the APRQ by Krystal et al. (1986) to distinguish the use of the items as a structured interview designed to be an objective measure of alexithymia.

Alexithymia—a difficulty with identifying and describing emotions—has become an important focus of research in understanding various aspects of autism. Early studies by Berthoz & Hill (2005), Hill et al. (2004) and Liss et al. (2008), established higher rates of co-occurring alexithymia in ASD samples. Subsequent studies by researchers such as Silani et al. (2008) suggested that it is not a lack of physiological arousal associated with emotion that differentiates alexithymia, but rather difficulties at the second-order level of interoception—the conscious awareness of this arousal. Interoception refers to a complex range of processes through which an individual senses, interprets and integrates signals originating in the body (Khalsa et al., 2018). The link between alexithymia and autism may be mediated by subjective interoceptive impairments, with evidence of higher correlations between measures of interoception deficits and alexithymia in ASD samples than for the general population (Trevisan et al., 2019).

Bird et al.’s (2010) work argues that alexithymia may account for lower levels of empathy seen in autism, which was previously thought to be due to cognitive mind-reading difficulties, such as weak Theory of Mind skills. More recent work has extended our understanding of the role of alexithymia in ASD to include difficulties in emotion regulation (Gormley et al., 2021; Milosavljevic et al., 2016) and attempts to examine the psychophysiological mechanisms by which alexithymia presents in ASD, including the differing roles of blunting of emotional experience (sometimes labelled Type 1 alexithymia) and reduced identification and description of otherwise normal emotional experiences (Type 2 alexithymia) (Gaigg et al., 2018).

Some researchers have argued the exclusive use of self-report measures to assess alexithymia is counter intuitive (Gaigg et al., 2018; Trevisan et al., 2019; Waller & Scheidt, 2004). Alexithymia has been described as a problem of metacognitive monitoring (Vanheule et al., 2010). Metacognitions are “self-directed metarepresentational states” (Carruthers, 2014) and Gumley (2011) argued that deficits in metacognition can disrupt a person’s self-experience, which has significant implications in the study of alexithymia. Moreover, given the potential overlap between alexithymia and impaired metacognitive capacities, caution is needed when interpreting self-report measures. As Murphy and Lilenfeld (2019) point out, some individuals may not have the requisite metacognitive ability to gauge and report on their own mindreading ability, which according to some accounts, are required for self-awareness of one’s own emotional states (Carruthers, 2009). However, Lane et al. (2015) suggested that alexithymia, as measured by the TAS-20, depends to some degree on an awareness of one’s own impairments in emotion identification and description, as it is based on self-report, but also point out that this awareness may be more present in ASD, as people with ASD are more likely to have had these deficits highlighted by others, and retained this as semantic knowledge.

One method to tap alexithymia without the use of self-report is the APRQ. Participants are given 17 statements about imaginary scenarios and encouraged to report on how they might feel. Responses are assessed for their emotional content, and are scored on a binary scale. The summation of the ratings for all items gives an overall level of alexithymia. Krystal et al. (1986) recommended scoring responses as alexithymic if they do not mention affect. The most common alexithymic responses focus on actions, detailed descriptions of the imagined scenario, or descriptions of physical sensations. The APRQ was found to highly correlate with the BIPQ (Krystal et al., 1986) and was described as being a promising measure of alexithymia in early studies, since it is easy and quick to administer (Kosten et al., 1992), had high test–retest reliability (Kosten et al., 1992) and had excellent inter-rater reliability for the total score (Krystal et al., 1986), though it has rarely been used (Ryan et al., 2020). However, an additional advantage of the APRQ, is that is provides verbal samples from participants to standardised emotion-inducing scenarios.

A number of studies have attempted to clarify the relationship between verbal emotional expression and the alexithymia construct. A common strategy has been to compare expressions of emotional content with subjective measures of alexithymia such as the Toronto Alexithymia Scale (TAS-20). As pointed out by Roedema & Simons (1999) we might expect participants with high alexithymia to demonstrate impoverished affective self-description, even if their judgements of emotional stimuli are not impaired. Roedema & Simons (1999) found that participants with high levels of alexithymia produced fewer affect words than the participants with lower alexithymia, in response to a selection of slides from the International Affective Picture System. Similarly, Stone & Nielson (2001) investigated the relationship between verbal emotional expressiveness and scores on the TAS-20 with open ended questions on the assumption that high alexithymic participants would use fewer affect words. However, in contrast to Roedema & Simons (1999), they found no significant differences between a low alexithymia and high alexithymia sample, in either percentage or total number of affective words used in either experimental condition. Limitations of the study include that it was not clear what proportion of the “high alexithymic” student sample actually scored above the commonly used clinical cut-off of 61 on the TAS-20 total score and secondly they did not report on their method for defining words as affective or not, other than by reference to the method employed by Taylor et al. (1981) which involved defining affect words as those that “clearly and unambiguously expressed emotional feeling”. Friedman et al. (2003) in a study of adults found participants with ADHD, who had significantly higher levels of alexithymia than the control group, used significantly fewer affective words when describing scenes from film clips with strong emotional content, than used by the controls. Whereas a study by Tull et al. (2005) found that, contrary to their expectations, higher scores on the *Difficulty Identifying Feelings* subscale of the TAS-20 were associated with more negative emotion word use, but with less frequent use of words with positive affect. However, this study relied on the use of a procedure in which participants were prompted to talk about a distressing event in particular, which may have had an impact on the kinds of associations that emerged. In contrast, Wagner and Lee (2008) had participants talk about both positive and negative events in two separate experimental conditions—alone, or with another person, and in both cases found strong inverse correlations between TAS-20 total and ratings of verbal emotional expressiveness that were congruent with the valence of the talk. That is, alexithymia was associated with less negative expression during negative topics and less positive expression on positive topics.

Much of the inconsistency in results from these various studies may stem from the wide and disparate range of methods employed (Wagner & Lee, 2008). This is particular the case with the tendency to rely on idiosyncratic stimuli for the generation of emotional responses, such as viewing specific (but unspecified) emotional scenes from commercial movies (Friedman et al., 2003), or prompting participants to talk about autobiographical events, which inevitably vary between individuals (Wagner & Lee, 2008) or watching video of oral surgery (Stone & Nielson, 2001). Furthermore, caution is needed when interpreting these findings, as many of these studies used samples with few participants meeting the clinical threshold for alexithymia on the TAS-20 (Friedman et al., 2003; Rief et al., 1996; Stone & Nielson, 2001; Wagner & Lee, 2008).

A second methodological concern is how one establishes whether the words used by participants in a study are verbal expressions of emotion, beyond stating that they are clear and unambiguous (Taylor et al., 1981). In an attempt to reduce the subjectivity of raters and increase reliability, some researchers have used computerized approaches to the measurement of affective language use, employing linguistic analysis software such as the Linguistic Inquiry and Word Count (LIWC; Pennebaker et al., 2015), which has become the most widely used software for analysing text and narratives in clinical psychology (Alpers et al., 2005), not least because of the strong focus on dimensions associated with emotional and psychological processes (Fung et al., 2017). LIWC software uses a word counting strategy to categorise words as belonging to a broad affective word group, positively or negatively valanced categories, and to further sub-groups such as words associated with specific emotions (e.g. Anxiety, Anger, Sadness) and returns the percentage of words in each narrative that belong to each word category. Differences in proportion of emotion words used between high and low alexithymic individuals does not appear to be due to a general deficit in accessing emotional vocabulary, but rather a difference that emerges in response to affect-laden stimuli (Luminet et al., 2004). However when these differences emerge, they sometimes vary according to the valence of the emotion: the difficulty describing feelings subscale of the TAS-20 was found to correlate negatively with the proportion of positive emotion words used but not negative words (Paez et al., 1999).

Many researchers have argued that alexithymia research would benefit from techniques that move beyond self-report, to incorporate the ratings of others and the analysis of alternative data sources (Kooiman et al., 2002; Leising et al., 2009; Ricciardi et al., 2015). Additionally, the hypothesised link between language and emotion processing abilities has been widely discussed in the alexithymia literature (Hobson et al., 2019; Welding & Samur, 2018) however few studies have empirically investigated this connection.

In this study we analysed data from an earlier investigation (x, 20..) to explore the potential differences in affective language use between samples with and without autism. Given the risks of prejudging alexithymic status of participants on the basis of the TAS-20 self-report, in this study we examined differences between affective word use in ASD and non-ASD samples, rather than samples defined by TAS-20 scores. This allows for a more exploratory approach of comparing emotion language use across samples, rather than assuming that the TAS-20 will clearly identify all ASD participants with co-occurring alexithymia or low emotional language use.

First, we expected the neurotypical (NT) sample to use more emotion words in general than the ASD sample, and we expected this difference to hold even as a proportion of total words spoken. Secondly, we predict that higher emotion word use will correlate with lower scores on the AQ10 as a measure of ASD symptomology. Furthermore, responses of ASD participants to the emotional provocation may differ to those of the NTs through incongruous emotional responses. Thirdly, if the alexithymia in ASD is independent of general verbal expressiveness, we expect that no difference in verbal expression will be noted between the samples on the measurement of overall word count.

A simple emotional word count does have some limitations. It does not distinguish between participants with a fine grained emotional awareness and those who use a narrow range of emotion words, but who use them with a high frequency. Vine et al. (2020) developed a measure of emotion vocabulary (EV) by counting the rate of distinct, non-repeated emotion words within a transcript or interview, which measures the diversity of emotion terms used rather than simply the frequency. This is calculated as the proportion of unique emotion words as a percentage of total word count (after the removal of stop words). Fourthly, given the risk that EV may represent a more general verbal ability, a point made by various previous researchers (Hobson et al., 2019; Luminet et al., 2004), the type/token ratio can be calculated for each participant as a measure of general linguistic ability. This is the number of unique words divided by the total number of words used. We use a similar method to Vine et al. (2020), calculating the type/token ratio after the removal of stop words. However, we did not remove EV words as, for some respondents, emotion words make up a considerable portion of their responses, because of the nature of the APRQ questions being solely focused on high emotion scenarios. We predicted the ASD sample will score significantly lower than the NT sample on the EV measure, but that no such difference will emerge on the type/token ratio.

Fifthly, the bag-of-words approach (Silge & Robinson, 2017) so far outlined, may be at risk of missing some more complex emotional expression, such as those that take the syntactic form "I feel…". To address this, all bigrams of the "feel…" construction were examined and tested for differences between the samples. Finally, to establish if emotional word use distinguishes between the samples, over and above the predictive power of the TAS-20, we carry out a logistic regression, with TAS-20, emotional and non-emotion word use, as predictors of group membership.

## Method

### Participants

A total of 64 participants were recruited to take part in this study, 32 with a diagnosis of ASD (mean age = 26.5 years) were recruited from an ASD support service that requires a full diagnostic work up by a clinical team before accessing the service. Diagnostics are carried out using the Autism Diagnosis Interview-Revised (ADI-R) (Lord et al., 1994) or the Diagnostic Interview for Social and Communication Disorders (DISCO, Wing et al., 2002), and the Autism Diagnostic Observation Schedule (ADOS; Lord et al., 2000). For the neurotypical (NT) sample 32 participants (mean age = 24.5) were recruited through various advertising campaigns within university, social media and word of mouth and had the same sex distribution as the ASD sample (25 male, 7 female). The groups did not differ significantly in age (*t*(59.58) = 1.06, *p* = 0.295, *d* = 0.27 however, levels of education differed significantly between the two samples χ2(4) = 10.11, *p* = 0.03. The NT sample had a higher proportion of graduates (66%–41%). In both samples 31 out of 32 participants identified their nationality as Irish.

## Measures

### Autism Quotient (AQ-10)

Autism traits were measured using the 10-item version of the AQ scale which is made up of 10 descriptive statements about preferences and habits, answered on a four-point Likert scale. A bimodal scoring system is used where responses are scored as either one or zero by merging each of the two levels of disagree and agree (AQ-10 Allison et al., 2012). This results in a potential score of between 0 and 10. For screening, six or more positive responses to the bimodal scoring is used (Allison et al., 2012). The AQ-10 had very good internal consistency in our samples (α = 0.96) higher than the value (α = 0.85) reported in the original AQ-10 study by Allison et al. (2012).

### Alexithymia Provoked Response Questionnaire (APRQ)

The APRQ is a 17-item structured interview whereby participants are asked to imagine various emotional states in response to a range of hypothetical scenarios. Responses are then evaluated by the interviewer based on the participants ability to identify and describe emotions and emotional experiences (Kosten et al., 1992). Each response is rated as being either 1 or 0 with a higher score in the APRQ being indicative of a lower degree of alexithymia and a lower score indicating higher degrees of alexithymia. Responses were scored as alexithymic if they described an intended action but not affect e.g. “I’d run” or “I’d fight”, contained descriptions of physical sensations rather than affect or described the situation rather than affect (Kosten et al., 1992).

### Toronto Alexithymia Scale (TAS-20)

The Toronto Alexithymia Scale (TAS-20) is a twenty item self-report measure of alexithymia, developed by Bagby et al. (1994). All items are rated on a five-point Likert scale from strongly disagree to strongly agree. A total score is the sum of the 20 items. In the original study, the TAS-20 demonstrated acceptable internal consistency with Cronbach’s α estimate of 0.81 for the total score. In our samples, the overall Cronbach’s α estimate was 0.81 for the total score. In the TD sample, 5 participants scored above the cut-off of ≥ 61 for identifying alexithymia, representing 15.6% of the TD group. In the ASD sample, 18 of the participants or 56.2% were in the alexithymic range.

### Text Analysis

Following a study by *the current authors* (20x) a secondary data analysis of affective vocabulary use among participants was carried out using two methods: the computer software ‘Linguistic Inquiry Word Count’ (LIWC; Pennebaker et al., 2015) and a tidytext approach utilising the R package "tidytext" (Silge & Robinson, 2017).

#### LIWC

The Linguistic Inquiry and Word Count (LIWC) program is a widely used automated text analysis tool, which can analyse a selection of either texts or transcripts of audio recordings of natural conversations (Iliev et al., 2015; Mehl, 2006) and calculate the percentage of word counts from a variety of semantic categories, such as *affective processes, social processes* and *cognitive processes* (Tausczik & Pennebaker, 2010). It includes psychologically meaningful categories such as positive and negative emotions, overall affective word usage as well as specific counts of individual emotions such as joy, anger, sadness and fear (Pennebaker et al., 2015). It has been used extensively in a wide variety of psychological studies, such as of narcissism (Holtzman et al., 2019), trauma (Borelli & Sbarra, 2011), ADHD (Guntuku et al., 2017) and ASD (Nguyen et al., 2013). Our analysis focused on comparing overall emotional word usage across groups, comparing positive and negative emotions as well as emotion type analysis across groups (for instance, comparing responses to anxiety provoking questions).

### Emotion Vocabulary

An alternative linguistic analysis technique to the use of proprietary software, is the tidytext package (Silge & Robinson, 2017). This R package allows the user more direct access to the raw data when analysing texts, with the option to pick the most suitable lexicon for the analysis (Priyavrat & Sharma, 2018). The package comes with three sentiment analysis dictionaries, however, these are very diverse with between 2500 and 14,000 words in each. The sentiment lexicons are much broader than lists of emotional states, but rather include words and associated affects, for instance the NRC lexicon list the words “abject”, “gossip” and “parrot” as negative and “chocolate”, “gymnast” and “ripe” as positive. For the evaluation of language use in alexithymia and ASD, a lexicon is required that is more specific to emotional states. The OCC account of emotion (Clore & Ortony, 2013; Ortony et al., 1987) attempted to isolate the English language terms that identify emotions. They undertook a thorough and systematic review of the affective lexicon in English, and from 650 candidate words, they created a taxonomy of 248 affective states that met a set of specific conditions. Each word needed to describe an internal state and should be primarily focused on affect, as opposed to behaviour or cognition. We used these 248 words as an affective dictionary in this study. In the OCC lexicon, some emotion words are only included as adjectives (e.g. “angry”) and not nouns (“anger”), whilst the authors’ acknowledged that they only provide the adjectively form of words, even when there is little semantic difference. This would matter little in a close-reading methodology, but for computerised linguistic analysis, it is important that the lexicon is comprehensive enough to detect occurrences of emotions in alternate syntactic forms. We therefore supplemented the OCC lexicon with the syntactic variants of emotions where the semantic meaning is identical or near identical (for a full list of emotion terms used see appendix 1).

As a number of authors have pointed out, if alexithymia is unrelated to general language use, non-emotional language use should be unaffected, whereas if abnormalities are observed in non-emotional word use, this would suggest that alexithymia is, to at least some degree, mediated by a more general language impairment (Hobson et al., 2019; Luminet, et al., 2004). To test this hypothesis, we calculated the number and variety of both emotional and non-emotional words used by each participant in the study, after removing stopwords. We compared the number of total words per person across samples to investigate if the ASD and NT group differed in the total number of words used, emotion words used or non-emotion words used. We also correlated these three measures with the TAS-20 total score and APRQ scores.

To supplement this bag-of-words approach, we also utilised bigrams, tokenising the responses into two-word pairs, where the first word implied some emotional state such as "feel awful". The benefit of this approach is that it offers additional context that can be illuminating when examining sentiment (Silge & Robinson, 2017). For instance a person might report being "not happy", which as a bigram is clearly an indicator of negative sentiment, but which when tokenised as a unigram ("happy") has the opposite valence.

### Data Analysis Strategy

Skewed distributions are common in linguistic analysis studies, due to the occurrence of Zipf’s law and Heap’s Law, therefore we evaluated most of the outcomes with nonparametric methods. When using the Wilcoxon rank sum exact test, we calculated the effect size using Vargha and Delaney’s method (Vargha & Delaney, 2000), which allows for a standardised quantification of between group difference, in which a value of 1 indicates complete stochastic dominance of the first group over the second and a value of 0 represents the opposite. Therefore, values of 0.5 or close to, indicate no significant differences between groups. As the values of the Vargha and Delaney’s *A* statistic can go in either direction of 0.5, ranges are given interpretation guides as follows. The interpretation of effects sizes by Vargha and Delaney are: negligible effect: 0.45–0.55; small effect: 0.56–0.63 or 0.35–0.44; medium effect: 0.64–0.70 or 0.30–0.34; large effect: > 0.70 or < 0.30.

## Results

### LIWC

ASD participants used significantly fewer affective words (mean = 11.25, SD = 6.88) than NT participants (mean = 16.53, SD = 9.62), *W* = 297, *p* = 0.004, *A* = 0.29 (values less than 0.3 indicate a large effect size). ASD participants also used significantly fewer negative affective words (mean = 7.47, SD = 6.66) than NT participants (mean = 12.36, SD = 8.79), *W* = 263, *p* = 0.001, *A* = 0.257 (large effect size), however the difference between groups on positive affective word use was not significant *W* = 449, *p* = 0.40, *A* = 0.438 (negligible effect size). Positive emotions constitute a much small proportion of the affect words used overall by all participants, in part due to the lack of focus in the APRQ on positive scenarios (only two questions posit positive events—being complimented in two different ways), therefore the power to detect significant differences between the groups is much weaker for positive emotions.

**Fig. 1:**
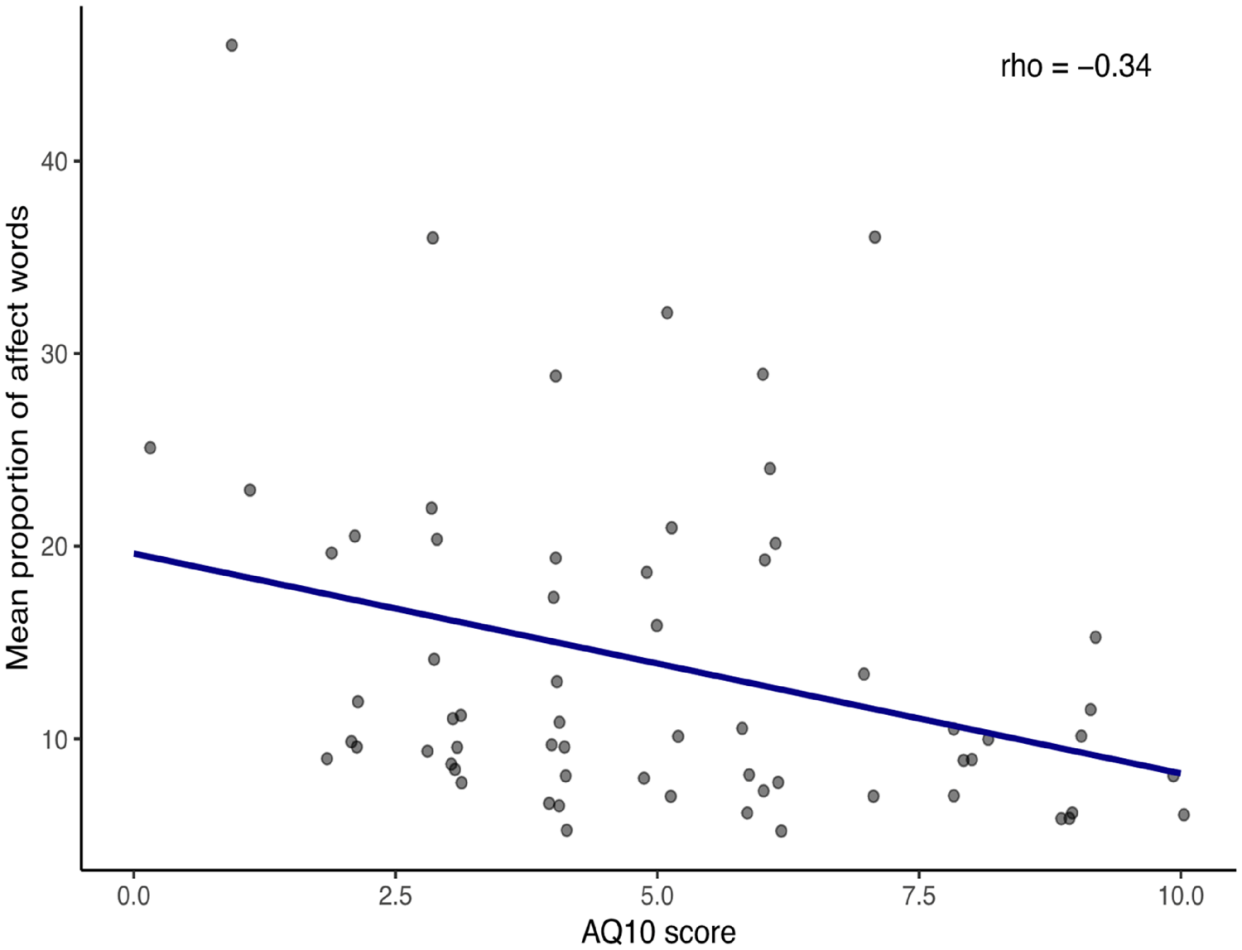
Correlation between AQ10 scores and mean proportion of affect words used

There is a significant correlation between higher ASD symptoms measured by the AQ10 associated with lower proportions of affective words used, rho(62) = − 0.34, *p* < 0.006 (see Fig. 1).

However, there was no correlation between TAS-20 total score and the proportions of affective words used, rho(62) = − 0.12, *p* < 0.36.

As the APRQ features a range of different emotion provocations, we can examine the kinds of affect words used that are congruent with the provocation. Six questions are likely to provoke anger (items 2, 3, 5, 6, 9, 10) featuring scenarios of injustice and insults. Four items feature fear-inducing scenarios (items 4, 8, 14, 15) that centre on threats to the person’s life, that one might predict would produce anxious words. One question is about loss (item 13) and is likely to produce a higher frequency of sad words. We filtered the dataset by the kind of provocation and for each one we calculated the mean use of LIWC emotion categories that were congruent. As can be seen in the Table 1, in each of the three emotion categories from the LIWC, the ASD participants produced significantly smaller proportions of congruent affective words.

**Table 1:** Mean emotion word proportions by emotion type and provocation type

Provocation	LIWC category	ASD Mean (SD)	NT Mean (SD)	t-value	*p* value	Effect size
Anger	Anger	4.02 (5.00)	8.58 (7.36)	− 2.901	0.005	− 0.73
Anxiety	Anxiety	6.70 (10.98)	14.43 (14.27)	− 2.426	0.018	− 0.61
Sad	Sad	3.41 (6.79)	9.58 (15.81)	− 2.029	0.049	− 0.51

We examined whether the ASD participants produce more incongruent emotional responses to the various forms of emotional provocation on the APRQ. There were no statistically significant differences between the groups for incongruous word use to anger provocations, with sad responses t(291.62) = − 1.71, *p* = 0.09 and anxious responses t(376.09) = − 0.79, *p* = 0.43. There were no statistically significant differences between the groups for incongruous word use for sad words in relation to the anxious provocations, t(291.62) = 0.58, *p* = 0.57, however, there was a significant difference between the groups for angry word responses to anxious provocations, t(170.59) = − 2.16, *p* = 0.006, d = − 0.27 with the NT group producing significantly more words relating to anger in response to anxious scenarios than the ASD group. Finally, in relation to sad provocations, the number of anxious responses was not significantly different t(291.62) = 1.20, *p* = 0.24, but the NT group produced significantly more angry words *t*(376.09) = − 2.93, *p* = 0.006, d = − 0.73.

In summary, the groups did not differ in their use of incongruent emotional words to anger provocations, but the NT sample did produce higher proportions of anger words to anxious provocations and sad provocations than the ASD group.

### Emotion Vocabulary (tidytext)

**Fig. 2:**
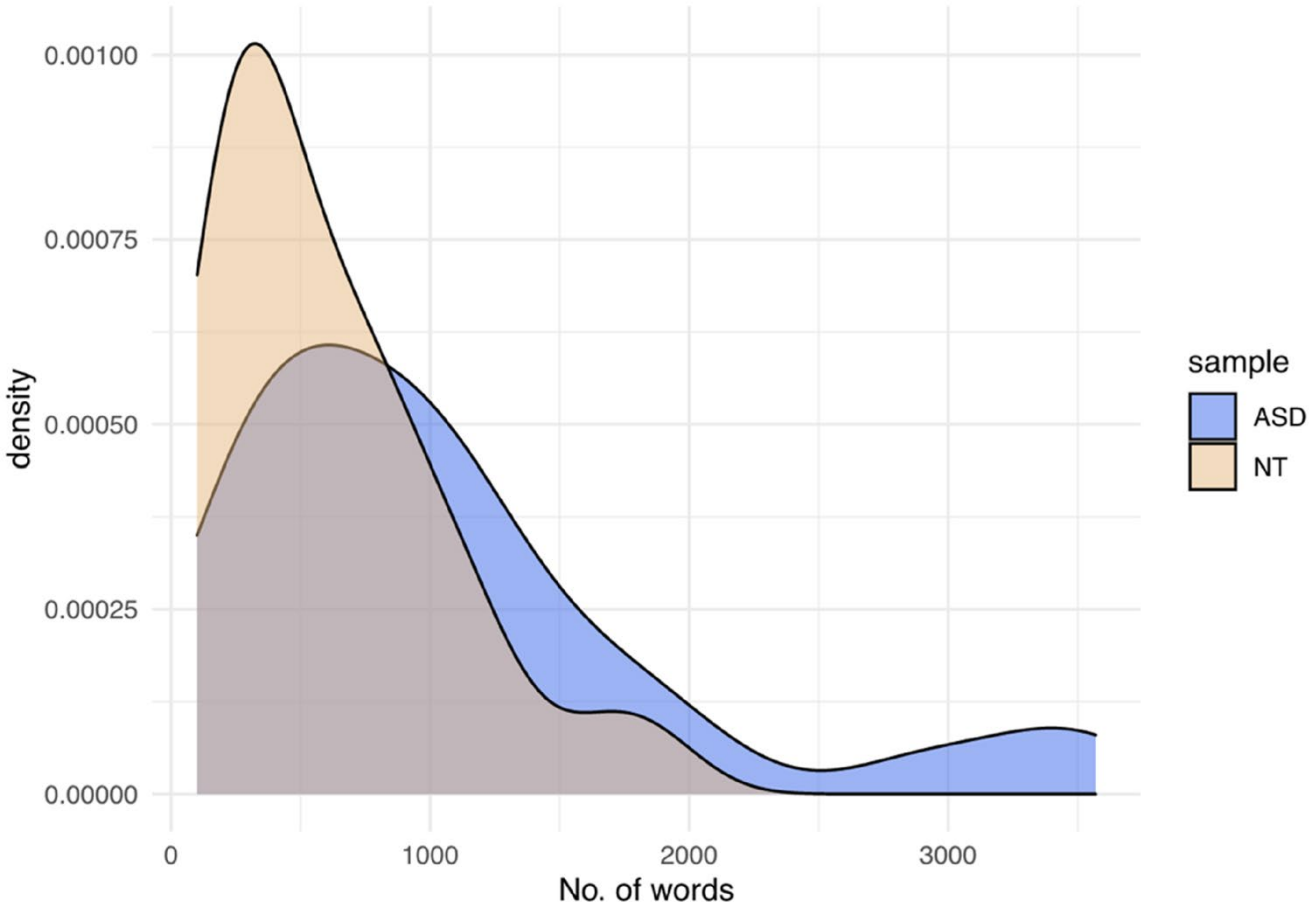
Density plot of total raw word count for each sample

Once all of the interviews were tokenised, we calculated the total number of words used by each participant, before the removal of stop words. Word counts for the two samples differed significantly, with ASD participants on average producing longer responses per interview (M = 1066 words, SD = 877) compared with NT participants (M = 606 words, SD = 442), *W* = 688.5, *p* = 0.02. Though, as the density plot reveals in Fig. 2, there is considerable overlap between the distributions—evidence of similarities that should not be overlooked (Hanel et al., 2019).

We used the stopword collection provided by the tidytext package, with the addition of a custom stopword filter that included the transcribed filler sounds such as "hmm", "ahm", “ehm”. The removal of stopwords reduced the difference between samples on word count, with ASD (M = 209, SD = 168) and NT participants (M = 134 words, SD = 87) no longer differing significantly in the number of words used per interview (*W* = 647, *p* = 0.07). We found no relationship between word count and alexithymia as measured by the TAS-20, *r*_*s*_ = 0.02, *p* = 0.87, or between word count and alexithymia as measured by APRQ, *r*_*s*_ = − 0.18, *p* = 0.15. After removing stop-words, we also calculated the use of distinct words per interview for each participant as a measure of their range of vocabulary. ASD participants used a wider range of distinct words (M = 131, SD = 96) than the NT participants (M = 85.66, SD = 57.1), *W* = 666, *p* = 0.04, confirming that ASD participants do not lack a general verbal expressivity.

In addition to unique emotion words used per participant, using a similar method to Vine et al. (2020), we also calculated the EV scores, based on the OCC lexicon. Table 2 shows that the ASD sample used fewer emotion words overall than the NT group, though this difference did not quite reach statistical significance and the effect size was small. However, the EV scores for the NT sample was significantly higher than the ASD sample, with a medium effect size, whereas no such differences emerged in the type/token ration (TTR), as a measure of general verbal ability. The use of non-emotion words differed significantly between the samples with the ASD group using significantly more non-emotion words than the NT group, with a medium effect size.

**Table 2:** Emotion word count, EV, TTR and non-emotion word count by sample

	ASD	NT	*p* value	Effect size (VD.A)	Effect size
Emotion word count	24.09 (11.87)	28.66 (11.77)	.06	0.36	Small
EV	8.24 (6.01)	11.84 (7.78)	.01	0.312	Medium
TTR	65.19 (8.67)	63.44 (8.23)	.55	0.543	Negligible
Non-emotion word count	184.62 (157.60)	104.63 (79.69)	.02	0.67	Medium

The correlations between EV, Emotion word count and TTR, with AQ10, TAS-20 and Age were all examined. As can be seen in Table 3, the AQ10 score was negatively correlated with the Emotion Vocabulary score, and this remained significant even after controlling for the Type/Token Ratio, with a partial correlation analysis, *rho*(62) = − 0.28, *p* = 0.05. Unsurprisingly, there was a strong relationship between APRQ and the EV score, and an even stronger relationship between APRQ and the Emotion Word Count. The Type/Token Ratio was also negatively correlated with the APRQ. Age was negatively correlated with EV alone.

**Table 3:** Zero-order correlation between variables

Variables	EV	TTR	Emotion word count	APRQ	Age	TAS-20
AQ10	− .29*	.05	− .08	− .42***	.11	.47***
TAS-20	− .10	− .01	-.10	− .19	− .03	
Age	− .32**	.03	.04	− .26		
APRQ	.43***	− .53***	.62***			
Emotion word count	− .17	− .60***				
TTR	–.01					

*p < .05, **p < .01, ***p < .005

To check for emotional word use not reflected in the bag-of-words approach, bigrams were calculated which had the first of the two words as either ‘feel’, ‘feels’ or ‘feeling’. In total there were 195 matches for bigrams with the most common occurred with *feel* words were ‘feel like’ (54 occurrences), ‘feel a’ (41 occurrences) ‘feel I’ (29 occurrences). After removing stopwords from the bigrams, 95 matches remained. Instances when “feel” is followed by an emotion such as “sad” probably tells us little more than the emotion word counting we have already done with the OCC lexicon and EV calculation, so we filtered out instances of ‘feeling—(OCC) emotion word’. This left a small number of occurrences of ‘feel hungry’, ‘feel sick’ and ‘feel bad’, but the remaining occurrence never appeared on more than two occasions, which demonstrates that there is little evidence emotions are being expressed with a “feel —” formation, other than those already identified with the OCC lexicon.

### Logistic Regression

Finally, to assess the degree to which affective word use differentiates between the ASD and NT samples over and above TAS-20 scores, we conducted a logistic regression, with diagnostic status as the dependent variable. In the first model, (see Table 4) TAS-20 significantly improved correct classification over base rate predictions with the rate of correct classification rising from the base rate of 50.79% (this was not 50% as only 63 participant completed the TAS-20), to 68% with the TAS-20. We compared this model using a logistic regression of the TAS-20 and two linguistic based predictors: the total emotion word count (tokens) and the total non-emotional word count. To avoid the risk of multicollinearity, composite scores, such as the EV and TTR, were not included as predictors. This model improved predictions over the TAS-20 alone model with the rate of correct classification rising from the TAS-20 rate of 68%–81% with the inclusion of the two linguistic variables. As the odds ratio for emotional word count is less then 1, increased emotional word count is associated with lower likelihood of being in the ASD group, whilst the odds ratio for non-emotional word use is greater than 1, indicating that the high frequency of non-emotional word use is associated with a higher likelihood of being in the ASD group.

**Table 4:** Logistic regression with ASD group membership as outcome variable

	*b*	*z statistic* (Wald)	Odds ratio (95% CI)	*R*^2^	AIC	Model
TAS-20 Model						
Constant	− 4.93 (1.56)	− 3.16		0.19 (Cox & Snell)		
TAS-20	0.09 (0.03)	3.22	1.09 (1.04–1.16)	0.26 (Nagelkerk)	77.74	*X*^2^(1) = 13.58***
TAS-20 and linguistic model						
Constant	− 4.39 (2.14)	− 2.05				
TAS-20	0.11 (0.04)	2.85	1.11 (1.04–1.20)			
Emotion word count	− 0.15 (0.05)	− 3.05	0.86 (0.77–0.94)	0.42 (Cox & Snell)		
Non-emotional word count	0.02 (0.01)	3.05	1.02 (1.01–1.03)	0.56 (Nagelkerk)	60.77	*X*^2^(3) = 34.56***

*CI* confidence interval

***p < .001

The Hosmer and Lemeshow Goodness-of-fit test gave a Chi-squared value 6.31 for the second model, which was not significant (χ^2^(8) = 6.31 *p* = 0.61, NS), indicating the model is an adequate fit of the data. The Wald *z*-statistics in Table 4 indicates that both emotional word count and non-emotional word count are at least as important as predictors of group membership as the TAS-20 score.

We conducted test diagnostics, finding that the VIF test resulted in scores below 5 for all three predictors, confirming low levels of multicollinearity. We also checked the linear assumption for logistic regression, between the logit transformation of the response variable and each predictor variables, using the Box-Tidwell procedure—none of the interaction terms was significant, indicating the assumption was justified. Cook's distances did not exceed 0.5, indicating little influence of large residuals or high leverage points.

## Discussion

This study explored the use of emotional language by NT and ASD participants while being interviewed with the APRQ. As predicted, ASD participants used significantly fewer affective words, and this reduction in emotional term use was strongly correlated with AQ10 score, but not related to TAS-20 scores. This confirms that, though the TAS-20 undoubtable measures one aspect of alexithymia, other differences in emotional responding present in ASD may not be detected by it. ASD participants used less affective words across a range of emotions, including anger, fear and sadness. Furthermore, the differences between samples could not be accounted for by incongruous emotional responses—on the whole, ASD participants used emotion terms much less frequently, but not incongruously.

Concerns have been expressed by a number of researchers that alexithymia may reflect a more general reduction in expressivity or difficulties accessing a neutral lexicon (Luminet et al., 2004; Roedema & Simons, 1999; Wotschack & Klann-Delius, 2013). In contrasts, ASD and NT participants did not did not differ significantly in their use of type/token ratios in this study, and in fact, ASD participants had a wider range of distinct words than the NT participants. On EV scores—the ratio of unique emotion words to total number of words used—ASD participants scored significantly lower than NT participants and the EV score correlated strongly with AQ10 and APRQ. Additionally, the logistic regression showed that emotional word count and non-emotional word count improve our ability to distinguish between ASD and NT participants beyond that found in the TAS-20. This provides clear evidence of an emotion specific difference in the performance of the ASD participants. It highlights two different kinds of emotional expressive difficulty present in ASD—both with the range of types of emotion identified, and with the frequency of use of emotion tokens. It is suggestive that participants with ASD may lack "emotional granularity" (Barrett, 2004; Kimhy et al., 2014) which is the degree to which individuals differentiate between different emotional states and can represent these precisely, and this may offer potential areas for intervention to improve social functioning.

A conceptual issue raised by the results is how do we interpret a participant who scores low on the TAS-20 and low on the emotional word use. Such a group was found in the current study, with a small proportion of low alexithymia individuals from the ASD sample, who also had very low emotional word count scores. We could interpret this to be evidence for ASD and co-occurring alexithymia that is not detectable with the TAS-20—alexithymia without self-insight potentially. The alternatively argument might be that elements of emotional expression difficulty exist in ASD that are not part of alexithymia. Though we find it difficult to reconcile this with the current definition of alexithymia, and are more inclined to regard the TAS-20 as an imperfect measure of the construct.

The majority of studies on ASD and alexithymia have utilised the TAS-20 to identify traits of alexithymia and as a measure of the strength of those traits. The current study highlights that, at least in terms of verbal emotional expressivity, the TAS-20 is not sensitive to some elements of alexithymia in ASD. The on-going concerns expressed by some researchers on an over-reliance on self-report measures in this area seems well founded (Gaigg et al., 2018; Kooiman et al., 2002; Ricciardi et al., 2015). Future research could extend the study of emotional verbal expressiveness by making a comparison with an alternative self-report measures of alexithymia such as the BVAQ (Vorst & Bermond, 2001) which includes more affective alexithymia subscales. Furthermore, the APRQ is limited in the range of emotions that it is designed to provoke, with a strong focus on negatively valanced emotions. There is scope for improving the design of this measure by including provocations of a wider range of emotions.

The current study has a number of strengths: the use of two different linguistic analysis techniques, a commercial software package (LIWC) and the R tidytext package, which produced results consistent with each other, suggests that the findings are robust. Secondly, many previous studies of emotional language use in alexithymia were not specific in how they define emotion words, however, the current study employed the OCC emotion lexicon (Clore & Ortony, 2013; Ortony et al., 1987), increasing the transparency of these judgements and making replication easier.

This study has a number of limitations. The bag-of-words approach does not allow one to check who the subject is of a specific grammatical emotion verb. As an example used by Clore et al. (1987), if “John hates Mary”, John is the both the subject of the verb, but also the one experiencing the emotion, but by contrast if we say “John irritates Mary”, it is the object of the verb—Mary, who is experiencing the emotion, not John. In a bag-of-words approach to linguistic analysis, no attempt is made to connect the emotion words used and the personhood of the one experiencing the emotion. It is possible that other differences occur in how participants attribute emotions to themselves or others, though the focus of the APRQ does mitigate this risk somewhat, with each of the seventeen questions being explicitly focused on the participant's own feelings. Furthermore, a more general limitation of the study is that despite the reasonable sample sizes, the exploratory nature of the study would benefit from replication with larger samples.

Overall, we demonstrated that emotional language use is much reduced in ASD participants, even when their scores on the TAS-20 do not indicate high levels of alexithymia. In conclusion, the APRQ, in combination with modern approaches to linguistic analysis afforded by the R programming language, opens the potential to a more sophisticated exploration of alexithymia in ASD, in a way that is not dependant on self-report measures. Emotional language use is an important difference in how participants with ASD self-narrate their experiences, and this area could form the basis of future interventions and supports to help reduce emotion dysregulation in ASD.

## Appendix

OCC emotion word list with variations

**Table:**

Admire
Admired
Admiring
Adore
Adored
Adoring
Affection
Affectionate
Afraid
Aggravate
Aggravated
Aggrieve
Aggrieved
Agitate
Agitated
Agitating
Agony
Aggravating
Alarm
Alarmed
Alarming
Amuse
Amused
Amusing
Anger
Angered
Anguish
Anguished
Annoy
Annoyed
Annoying
Anxiety
Anxious
Apathetic
Apologetic
Appreciated
Appreciating
Appreciation
Apprehensive
Approve-of
Ashamed
At-ease
At-peace
Attract
Attracted
Attracting
Aversion
Awe
Benevolent
Bitchy
Bitter
Blue
Broken-hearted
Burdened
Burdening
Calm
Calmed
Calming
Carefree
Charmed
Cheered
Cheerful
Cheerless
Comfortable
Compassionate
Concern
Concerned
Concerning
Console
Consoled
Consoling
Contempt
Content
Contented
Contrite
Cowardly
Crabby
Crush
Crushed
Crushing
Deflate
Deflated
Deflating
Deject
Dejected
Delight
Delighted
Delighting
Depress
Depressed
Depressing
Desire
Desired
Desiring
Despair
Despaired
Despairing
Desperate
Despise
Despised
Despising
Despondent
Detest
Detested
Devoted
Disappointed
Disappointing
Disapproved
Disapproving
Discontent
Discontented
Discourage
Discouraged
Disenchant
Disenchanted
Disgust
Disgusted
Disgusting
Dishearten
Disheartened
Disheartening
Dislike
Disliked
Disliking
Dismay
Dismayed
Dismaying
Displease
Displeased
Displeasing
Disprove-of
Disappoint
Dissatisfied
Dissatisfy
Dissatisfying
Distress
Distressed
Distressing
Disturb
Disturbed
Disturbing
Downhearted
Dread
Dreaded
Dreading
Eager
Ecstatic
Elate
Elated
Embarrass
Embarrassed
Embarrassing
Emotional
Empathic
Empathy
Encourage
Encouraged
Encouraging
Enjoy
Enjoying
Enjoyment
Enthusiastic
Envied
Envy
Envying
Euphoric
Exasperate
Exasperated
Exasperating
Excite
Excited
Exciting
Fear
Feared
Fearing
Fed-up
Fond
Forgive
Forgiving
Frighten
Frightened
Frightening
Frustrated
Frustrated
Frustrating
Fulfil
Fulfilled
Fulfilling
Fury
Gaiety
Glad
Glee
Gleeful
Gloomy
Glum
Grateful
Grief
Grief-stricken
Grouchy
Guilt
Guilty
Happy
Hate
Hated
Hating
Heart-stricken
Heartbroken
Hearten
Heartened
Heartening
Heartsick
Heartsore
Heavy-hearted
High
Homesick
Hope
Hoped
Hopeless
Hoping
Horrified
Horrify
Horrifying
Hostile
Humble
Humbled
Humbling
Humiliate
Humiliated
Humiliating
Hurt
Hurting
Ill-at-ease
Impatient
In-love
Incense
Incensed
Indignant
Infatuate
Infatuated
Insecure
Intimate
Intimidated
Irate
Irk
Irked
Irritate
Irritated
Irritating
Jealous
Joyful
Joyless
Joyous
Jubilant
Kind
Light-hearted
Like
Liked
Liking
Livid
Loathe
Loathed
Loathing
Lonely
Longed
Longing
Longing
Love
Loved
Lovesick
Loving
Low
Lust
Lusted
Lusting
Mad
Maddening
Malicious
Melancholy
Merry
Miserable
Mortified
Mortify
Mortifying
Mournful
Moved
Moving
Nervous
Nostalgic
Offend
Offended
Offending
On-edge
Optimistic
Outrage
Outraged
Outrageous
Overjoy
Overjoyed
Overwhelm
Overwhelmed
Overwhelming
Pained
Panic
Panicked
Panicking
Passionate
Peaceful
Peeve
Peeved
Pessimistic
Petrified
Petrify
Petrifying
Pine
Pined
Pining
Pissed-off
Pitied
Pity
Pitying
Placid
Please
Pleased
Pleasing
Pleasure
Proud
Rage
Raged
Raging
Reassure
Reassured
Reassuring
Regret
Regretted
Regretting
Relax
Relaxed
Relaxing
Relieve
Relieved
Relieving
Remorse
Repentant
Resent
Resented
Resenting
Respect
Respected
Respecting
Reverence
Sad
Satisfied
Scare
Scared
Scaring
Scornful
Secure
Self-conscious
Self-pity
Self-satisfied
Sensitive
Sentimental
Serene
Shaken
Shame
Shamed
Shaming
Shock
Shocked
Shocking
Shook-up
Shy
Sick-at-heart
Sicken
Sickened
Sickening
Smug
Solemn
Sooth
Soothed
Soothing
Sore
Sorrow
Sorry
Spiteful
Suffer
Suffered
Suffering
Suspense
Sympathetic
Tender
Tense
Tensed
Tensing
Terrified
Terrify
Terrifying
Thankful
Threatened
Thrill
Thrilled
Thrilling
Timid
Torment
Tormented
Tormenting
Touch
Touched
Touching
Triumphant
Troubled
Troubling
Uncomfortable
Uneasy
Unfulfilled
Unfulfilling
Unhappy
Upset
Upsetting
Uptight
Vengeful
Want
Wanted
Wanting
Warm
Woe-stricken
Wonder
Worry
Yearn
Yearned
Yearning
